# *TP53* germline mutations in the context of families with hereditary breast and ovarian cancer: a clinical challenge

**DOI:** 10.1007/s00404-020-05883-x

**Published:** 2020-11-27

**Authors:** Sabine Grill, Juliane Ramser, Heide Hellebrand, Nicole Pfarr, Melanie Boxberg, Christine Brambs, Nina Ditsch, Alfons Meindl, Eva Groß, Thomas Meitinger, Marion Kiechle, Anne S. Quante

**Affiliations:** 1 Department of Gynecology and Obstetrics, University Hospital Klinikum Rechts der Isar, Technical University Munich (TUM), Munich, Germany; 2 Institute of Pathology, Technical University Munich (TUM), Munich, Germany; 3 Department of Gynecology and Obstetrics, University Hospital of Augsburg, Augsburg, Germany; 4 Department of Obstetrics and Gynecology, Ludwig-Maximilians-University of Munich, Munich, Germany; 5 Institute of Human Genetics, University Hospital Klinikum Rechts der Isar, Technical University Munich (TUM), Munich, Germany

**Keywords:** *TP53* germline mutation, p53, Li-fraumeni-syndrome, Cancer surveillance, Breast cancer

## Abstract

**Purpose:**

*TP53germline (g)* mutations, associated with the Li-Fraumeni syndrome (LFS), have rarely been reported in the context of hereditary breast and ovarian cancer (HBOC). The prevalence and cancer risks in this target group are unknown and counseling remains challenging. Notably an extensive high-risk surveillance program is implemented, which evokes substantial psychological discomfort. Emphasizing the lack of consensus about clinical implications, we aim to further characterize *TP53g* mutations in HBOC families.

**Methods:**

Next-generation sequencing was conducted on 1876 breast cancer (BC) patients who fulfilled the inclusion criteria for HBOC.

**Results:**

(Likely) pathogenic variants in *TP53* gene were present in 0.6% of the BC cohort with higher occurrence in early onset BC < 36 years. (1.1%) and bilateral vs. unilateral BC (1.1% vs. 0.3%). Two out of eleven patients with a (likely) pathogenic *TP53g* variant (c.542G > A; c.375G > A) did not comply with classic LFS/Chompret criteria. Albeit located in the DNA-binding domain of the p53-protein and therefore revealing no difference to LFS-related variants, they only displayed a medium transactivity reduction constituting a retainment of wildtype-like anti-proliferative functionality.

**Conclusion:**

Among our cohort of HBOC families, we were able to describe a clinical subgroup, which is distinct from the classic LFS-families. Strikingly, two families did not adhere to the LFS criteria, and functional analysis revealed a reduced impact on *TP53* activity, which may suit to the attenuated phenotype. This is an approach that could be useful in developing individualized screening efforts for *TP53*g mutation carrier in HBOC families. Due to the low incidence, national/international cooperation is necessary to further explore clinical implications. This might allow providing directions for clinical recommendations in the future.

**Electronic supplementary material:**

The online version of this article (10.1007/s00404-020-05883-x) contains supplementary material, which is available to authorized users.

## Background

Germline (g) mutations in the *TP53* gene have rarely been reported in the context of hereditary breast and ovarian cancer (HBOC). So far, mutations have been associated with the Li- Fraumeni syndrome (LFS), an autosomal dominant cancer syndrome caused by a heterozygous germline mutation in the *TP53* gene. LFS predisposes to a wide spectrum of malignancies including premenopausal breast cancer, soft tissue and bone sarcoma, leukemia, brain cancer, bronchoalveolar lung cancer, and adrenocortical carcinoma (3). Some of the LFS-related cancers already occur during childhood or adolescence. For the diagnosis of LFS, the classic Chompret criteria must be met (Tables 1, 2) or a germline pathogenic variant in *TP53* is detected (3). At least 70% of individuals who meet the classic LFS criteria and approximately 20% of those, who meet the Chompret criteria have an identifiable germline pathogenic variant in *TP53* (3). The estimated lifetime risks of developing breast cancer for female *TP53* mutation carriers is 80–90% compared to 60–85%, for female *BRCA* mutation carriers [1, 2]. Until now, there has been no discussion on the recommendation of routine genetic testing for a *TP53g* mutation in families who fulfill the inclusion criteria for LFS, as shown in Tables 1, 2. In the general population, *TP53*g mutations are found at a very low heterozygous frequency of 0.025% [3]. However, recent data has shown that the introduction of next-generation sequencing has led to a considerably higher prevalence of *TP53*g mutations in the context of HBOC [2, 4].

**Table 1:** classic LFS criteria

Classic LFS criteria *(all criteria must be fulfilled)*
1. A sarcoma diagnosed before age 45 years AND
2. A first-degree relative with any cancer diagnosis before age 45 years AND
3. A first- or second-degree relative with any cancer diagnosis before age 45 years or sarcoma at any age

Adapted by Mai et al. 2012 [32]

**Table 2:** Chompret criteria

Chompret criteria for LFS
1. Proband diagnosed with a core LFS tumor (soft-tissue sarcoma, osteosarcoma, premenopausal breast cancer, CNS tumor, adrenocortical carcinoma) before age 46 years AND at least one first- or second-degree relative with a core LFS tumor (except breast cancer, if the proband has breast cancer) before age 56 years
2. OR a proband with multiple primary tumors (except multiple breast cancers), two of which are LFS core tumors, with the first occurring at age < 46 years
3. OR a proband with adrenocortical carcinoma, choroid plexus carcinoma, or rhabdomyosarcoma of embryonal anaplastic subtype, irrespective of family history
4. OR a proband with breast cancer < 31 years

Adapted by Bougeard et al. 2015 [28]

*LFS* Li-Fraumeni syndrome

In regards to clinical management of cancer patients, identifying a *TP53g* mutation is highly relevant when selecting a treatment regimen; studies suggest that mutation carriers are particularly sensitive to radiation exposure and chemotherapy, resulting in a substantially increased prevalence of secondary malignancies [1]. Breast cancer is the most commonly diagnosed cancer in *TP53* g mutation carriers, with risk estimates of 85% by age 60 [2, 3]. Indeed, in women diagnosed with breast cancer before age 40, there is a notable risk for a *TP53g* mutation; the prevalence varies from < 1 to 7%, whereas the highest risks are estimated for women with a breast cancer diagnosis before age 30 years [2, 4–7].

Recently, genetic testing for *TP53g* mutations has become more and more prevalent in clinical routine. However, it is challenging to counsel *TP53g* mutation carriers regarding the risk of hereditary breast and ovarian cancer. Among others, the multigene panel testing often reveals a variant of uncertain significance, whose relevance needs to be assessed prior to a potential translation into clinical practice. Elucidating possible genotype–phenotype correlations and other cancer-predisposing risk factors is inevitable in order to provide comprehensive genetic counseling.

This study aimed to further characterize the spectrum of germline *TP53g* mutations in a sample of 1876 well-defined familial BC/HBOC index patients from Germany who were tested negative for a germline *BRCA* mutation, and report on the mutation prevalence and clinical characteristics presented.

## Subjects and methods

### Study population

In the present study, we examined a cohort of 1876 HBC/HBOC index patients who were counseled and referred for genetic testing at the Department of Gynecology and Center for Hereditary Breast and Ovarian Cancer, Klinikum Rechts der Isar, Technical University of Munich (TUM) and Klinikum Großhadern, Ludwigs-Maximilians-University of Munich (LMU) between July 2014 and December 2017. 1464 patients were tested by panel analysis. 412 patients who tested negative for a pathogenic *BRCA* mutation respectively and met at least one of the inclusion criteria for genetic testing as proposed by the German consortium of Hereditary Breast and Ovarian Cancer (GC-HBOC) were evaluated by whole-exome sequencing (WES). The inclusion criteria are categorized as follows: A: breast cancer at any age with two or more close relatives with breast cancer at any age; B: breast cancer < 51 years. and at least one relative with breast cancer at any age; C: at least one breast cancer and one ovarian cancer; D: at least two women with ovarian cancer; E: at least one female and one male breast cancer; F: at least one ovarian cancer and one male breast cancer; G: early onset breast cancer < 36 years.; H: bilateral breast cancer (first disease diagnosed > 51 years.); I: a personal history of breast and ovarian cancer; J: triple-negative breast cancer < 50 years.; K: ovarian cancer < 80 years. (Table 3*)*.

**Table 3:** Stratification for subgroups by HBOC inclusion criteria for genetic testing and mutation prevalence

HBOC (*Hereditary breast and ovarian cancer*)-criteria	Total		*TP53* mut*		*TP53* VUS	
	*n*	*n* (%)	*n*	*n* (% )	*n*	*n* (%)
a. At least three woman with breast cancer independent of age	90	4.9	–		–	
b. OR at least two woman with breast cancer, one < 51 years	629	33.5	1	0.2%	14	2.2%
c. OR at least one woman affected by breast and one by ovarian cancer	173	9.2	–		–	
d. OR at least two woman affected by ovarian cancer	51	2.7	–		–	
e. OR at least one female and one male breast cancer	23	1.2	–		–	
f. OR at least one woman affected by ovarian cancer and one man affected by breast cancer	2	1.3	–		–	
g. OR at least one woman affected by breast cancer < 36 years	621	33	7	1.1%	5	0.8%
h. OR at least one woman affected by bilateral breast cancer, first < 51 years	209	11	2	0.9%	1	0.5%
i. OR at least one woman affected by breast and ovarian cancer	78	4	1	1.2%	–	

*TP53 mut: including (likely) pathogenic *TP53*g mutations

Within the counseling, a record of pedigree as well as medical report was provided for each patient including the age of diagnosis, histological subtype, tumor receptor status, tumor stage as well as personal and family history of cancer. Written informed consent was obtained from all patients. This study is approved by the ethics committee of the Technical University Munich.

### Analysis

Genomic DNA was isolated from blood samples of the patients. For 1464 cases, next-generation sequencing and data analysis were performed with an Illumina sequencing platform, using either the TruSight Cancer Panel (Illumina, San Diego, CA, U.S.) or the TruRisk Sequencing Panel (Illumina, San Diego, CA, USA). Target enrichment was performed using the TruSight Rapid Capture System (Illumina, San Diego, CA, USA). For 412 further cases, exome sequencing was performed with the Sure Select system for target enrichment (Agilent, Santa Clara, USA) and a HiSeq2500 system for sequencing (Illumina, San Diego, CA, USA).

All coding exons and adjacent intronic sequences (+ /− 20 bp) of the *TP53*-gene (NM_000546) were analyzed.

The variant classification was performed following the regulations of the GC-HBOC [8]. All variants were classified using a five-tier classification system (deleterious = class 5, likely deleterious = class 4, variant of uncertain significance (VUS) = class 3, likely benign = class 2, and benign = class 1).

For further evaluation, we exclusively focused on class 4 and 5 variants comprising missense, and essential splice-site variants as well as variants of uncertain significance (class 3) which had previously been verified by Sanger sequencing.

All *TP53* variants were proven to be germline by either segregation analysis or variant confirmation in corresponding non-cancerous breast tissue.

Statistical comparisons were performed using *IBM SPSS version 25*.

### Assessment

For functional assessment, we compared the *TP53g* variants according to two well-established published functional analysis patterns. First, an alignment with high-resolution mutation analysis by Kato et al. was enforced [9]. Stating that the sequence-specific transactivation is the critical function in *p53*-dependent tumor suppression, the working group used a comprehensive site-directed mutagenesis technique and a yeast-based functional assay to construct, express, and evaluate p53 mutants, and correlated p53 function with structure- and tumor-derived mutations [9]. Secondly, the synthetically designed *TP53* library created by Kotler et al. [10] was used to detect further coherences. The working group measured the functional impact of around 10,000 DNA-binding domain (DBD) p53 variants in human cells in culture and in vivo [10]. In order to allow quantitative comparison between variants, they calculated a relative fitness score (RFS) for each variant based on its retention (or depletion) and created a trained model that allows to provide an accurate estimation of the phenotypic effects of p53 variants [10]. Based on this model, Kotler et al. [10] suggested a RFS >  − 1 for *TP53* DBD mutations that compromise anti-proliferative functionality and correlated a RFS ≤ − 1 to *TP53g* mutations that retain anti-proliferative capacity [10]. They also suggest that LFS families with the six most prevalent hotspot mutations (R175H, R273H, R248Q, R248W, R273C, and R282W) even exhibit a somewhat lower age at tumor diagnosis by probably eliciting additional gain-of-function effects [10].

## Results

### *TP53g* mutation prevalence in the context of families with hereditary breast and ovarian cancer

Our analysis considered a total of 1876 index patients who presented with the diagnosis of breast cancer. In this cohort, the mean age at breast cancer diagnosis was 43 years (range 18–77). All corresponding pedigrees were conducted into seven mutually exclusive groups of clustered familial cancer histories (Table 3*)* according to the inclusion criteria of the GC-HBOC (Table 3). Overall, heterozygous (likely) pathogenic mutations in the *TP53* gene were detected in 11 of the 1876 familial female breast cancer index patients, yielding a prevalence rate of 0.6% (11/1876). The sequence variants comprised 10 missense mutations (10/11; 91%) and 1 splice-site mutation (1/11; 9%) (Table 5). The mean age of these *TP53g* mutation carriers at breast cancer diagnosis was 35 years (range 22–49 years). Clinical characteristics are summarized in *Supplement 1*. Eight of the *TP53g* mutation carriers presented with unilateral premenopausal breast cancer, whereas three women were diagnosed with bilateral breast cancer. Furthermore, two of the mutation carriers had a history of an adolescent malignancy (colorectal cancer 21 years; osteosarcoma 16 years.) in addition to their breast cancer disease. When considering the GC-HBOC inclusion criteria, the highest mutation frequencies were seen in families with at least one woman affected by breast cancer < 36 years. (1.1%) and families with at least one woman affected by breast and ovarian cancer (1.2%) (Table 3).

In addition, a total of 20 variants of uncertain significance (VUS) were identified in the *TP53* gene (Tables 3, 6), predicting a prevalence rate of 1.1% in the overall patient sample. Most VUS were rare missense variants (17/20; 85%). Indices carrying a VUS in the *TP53* gene presented with a mean age of 42.2 years (range 28–64 years.) at the time of breast cancer diagnosis. Clinical characteristics are provided in *Supplement 2*. The majority presented with unilateral breast cancer (*n* = 16), bilateral breast cancer was found in four women and there was no malignancy in childhood/adolescence. In this group, the highest mutation frequencies were found in families with at least two women with breast cancer, one < 51 years. (2.2%) (Table 3).

For a more detailed description of mutation frequencies, the familial cancer histories were further elaborated. The mutation prevalence in families with exclusive diagnosis of female breast cancer is shown in Table 4 (97.5% of all families). Group A comprises families with the exclusive occurrence of unilateral breast cancer (87.4% of all families), whereas group B (21.3%) primarily includes cases of premenopausal and postmenopausal bilateral breast cancer. (Likely) deleterious *TP53g* mutations and variants of uncertain significance were much more frequent in families with bilateral breast cancer compared to families with unilateral breast cancer (*TP53g mut*: 1.1% vs. 0.3%; *TP53g-VUS*: 1.4% vs. 0.9%, respectively) (Table 4).

**Table 4:** TP53 mutation prevalence in families with female breast cancer only

Familial cancer history (including proband)	Total *N*	% of total	*TP 53* mut* N	Prev %	*TP53* VUS *N*	Prev %
I. Total cohort	1876		11	0.6%	20	1.1%
II. GROUP A: female unilateral breast cancer cases only (bBC, mBC, and OC excluded)	1639	87.4%	5	0.3%	14	0.9%
III. GROUP B: female BC, of these > 1bBC (mBC and OC excluded)	366	21.3%	4	1.1%	5	1.4%

*bBC* bilateral breast cancer disease; *mBC* male breast cancer disease; *OC* ovarian cancer; *Prev* Prevalence

*TP53 mut: including (likely) pathogenic *TP53*g mutations

### Characterization of *TP53g* variants

#### Association of *TP53g* mutations with tumor receptor status

**Fig. 1:**
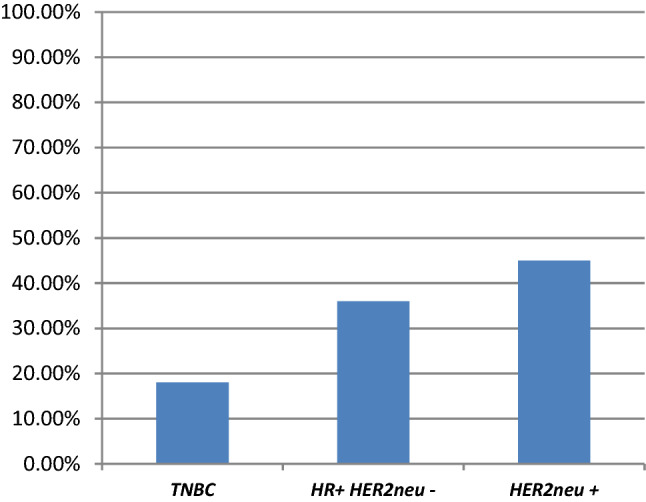
Distribution of tumor biology in (likely) deleterious TP53g mutations. The Figure depicts the phenotype of the tumors in this study, which was associated with a deleterious/likely deleterious TP53 mutation. *TNBC* triple-negative BC, *HR + HER2neu-* hormone receptor-positive HER2neu non-amplified, *HER2neu + * HER2neu amplified

Considering tumor biology, (likely) deleterious mutation carriers presented the following distribution at the time of the first breast cancer diagnosis: 18% TNBC (*n* = 2), 36% HR pos HER2-neg (*n* = 4), and 45% HER2-pos (*n* = 5) (Fig. 1*)*. Three out of eleven patients developed contralateral breast cancer within a median time of 2 years (range 0–4 years) from the first diagnosis. Tumor biology was equally distributed, exhibiting TNBC, HR pos HER2neg and HER2 pos biologies respectively.

### Alignment with LFS inclusion criteria

**Fig. 2:**
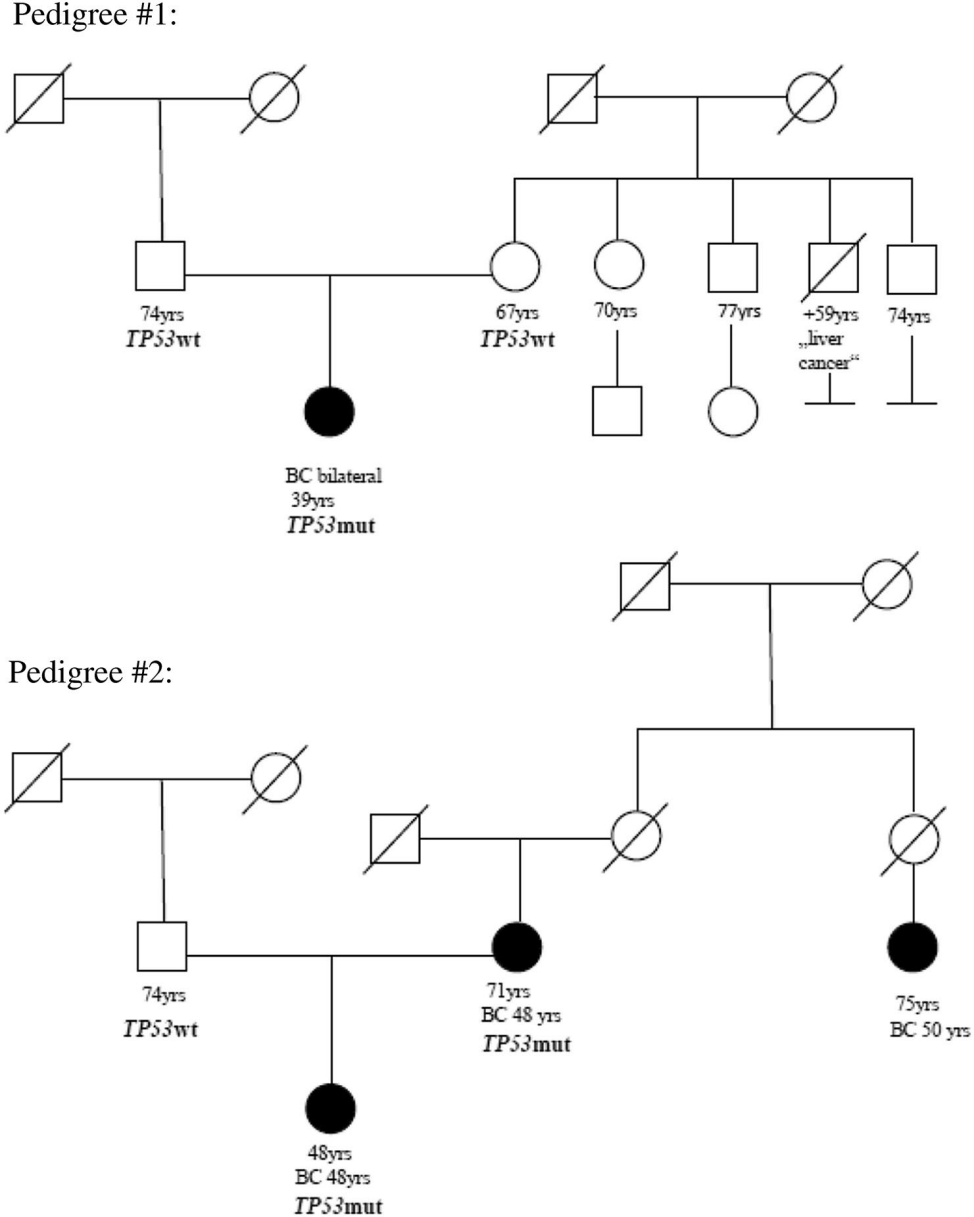
Pedigree#1 and #2 emphasize individuals with a pathogenic *TP53* germline mutation that lack classic personal or family history of LFS-related cancers and do not fulfill *TP53* testing criteria. Pedigree #1: Pedigree #1 displays a likely deleterious de novo *TP53* variant (NM_000546.5:c.375G > A (p.Thr125Thr)). The individual presented with a bilateral breast cancer disease at the age of 39 years. Pedigree #2: In pedigree #2 a likely deleterious *TP53* mutation (NM_000546.5:c.542G > A (p.Arg181His),) was detected, which was associated with the late manifestation of cancer disease. The index patient was diagnosed with breast cancer disease at the age of 46 years and her mother at the age of 41 years respectively

However, since LFS-families are typically categorized under the classic LFS/Chompret criteria, we additionally assigned the according pedigrees to these criteria. Interestingly, two of eleven patients with breast cancer disease had a (likely) pathogenic *TP53g* missense mutation and did not fulfill the LFS/Chompret criteria (18%) (*Supplement 1*). The corresponding pedigrees are shown in Fig. 2. The first patient carried a likely deleterious de novo* TP53g* variant (NM_000546.5:c.375G > A (p.Thr125Thr)) and presented with a bilateral breast cancer at the age of 39 years. (Fig. 2* pedigree #1*). The corresponding synonym variant is attributed to cause aberrant splicing [11–13] and has been reported in several LFS-families [12–18].

In another patient, the likely deleterious *TP53g* mutation (NM_000546.5:c.542G > A (p.Arg181His)) was detected, which has been shown to be associated with breast cancer in the literature, glioblastoma multiforme, and adrenocortical carcinoma in few individuals. However, a late manifestation of cancer disease (> 50 years.) was stated for all these patients, and functional analyses demonstrated mixed results regarding DNA binding, transactivation and growth suppression [10, 19–21]. Our patient was diagnosed with breast cancer at the age of 46 years, her mother at the age of 41 years (Fig. 2, pedigree #2). The mutation was confirmed to be germline by testing non-cancerous breast tissue of the index.

### Phenotype/genotype correlation by functional analysis

All (likely) deleterious mutations in this cohort are found to be located at the DNA-binding domain (DBD). Thus, no difference in DNA localization could be determined between corresponding pedigrees that fulfilled the classic LFS /Chompret criteria and those who only fulfilled the HBOC inclusion criteria.

For information on functional analysis, the transactivity level derived from the high-resolution mutation analysis by Kato et al. [9] could be obtained for nine of eleven (likely) deleterious *TP53g* mutations. The level of transactivity was strongly/ very strongly reduced in seven of these *TP53* mutations. One likely deleterious *TP53g* mutation revealed a moderate strong reduction (NM_00546.5:c.717c > G (p.Asn239Lys)) and only one likely deleterious *TP53g* mutation (NM_000546.5:c.542G > A (p.Arg181His)) showed a medium reduction of transactivity. This family did not meet the inclusion criteria for classic LFS (Fig. 2*, pedigree #2*).

Tumors in childhood and early adulthood were exclusively detected if p53 functionality was very strongly reduced. The same applied to family history.

The functional model by Kotler et al. [10] was applied to investigate for further coherences. We found seven of ten (likely) deleterious mutations of our cohort (*missing data: n* = *1*) to reveal a relative fitness score of > − 1, implicating a loss of proliferative functionality (Table 5) that was consistent with phenotypic characteristics. The corresponding pedigrees fulfilled not only the HBOC inclusion criteria but also the Chompret inclusion criteria. Interestingly, for pedigree 1 and 2, Kotler et al. [10] calculated a RFS ≤  − 1, which implies a retaining wtp53-like-anti-proliferative functionality and therefore explains the attenuated phenotype (Fig. 2).

**Table 5:** Functional data- (likely) deleterious mutations in the TP53 gene

Genomic position	Protein change	DNA domain	Transcriptional activity in yeast (% of wild-type)	Relative fitness score (RFS)**
c.375G > A	p.Thr125Thr	DNA-binding domain	Unknown	RFS ≤ − 1
c.542G > A	Arg181His	DNA-binding domain	Medium reduction (50.3%)	RFS ≤ − 1
c.700 T > C	Tyr234His	DNA-binding domain	Very strong reduction (0.89%)	RFS > − 1
c.717C > G	Asn239Lys	DNA-binding domain	Moderate strong reduction (26.4%)	RFS < −1
c.733G > A	Gly245Ser	DNA-binding domain	Strong reduction (4.2%)	RFS > − 1
c.742C > T	Arg248Trp	DNA-binding domain	Very strong reduction (0.09%)	RFS > − 1
c.743G > A	p.Arg248Gln	DNA-binding domain	Very strong reduction (0.21%)	RFS > − 1
c.783-2A > G splice acceptor	p.?	DNA-binding domain	Unknown	Unknown
c.800G > C	p.Arg267 Pro	DNA-binding domain	Very strong reduction (0.46%)	RFS > − 1
c.817C > T	p.273Arg > Cys	DNA-binding domain	Very strong reduction (0.76%)	RFS > − 1
c.818G > A	Arg273His	DNA-binding domain	Strong reduction (2.51%)	RFS > − 1

RFS ≤ − 1: retaining wtp53-like anti-proliferative functionality

RFS > − 1: disrupting functionality

Hotspot mutations: R175H, R273H, R248Q, R248W, R273C, R282W

**RTS (relative fitness score)

### Variants of uncertain significance

In a second step, we performed the same analysis for all 20 VUS, which occurred in our cohort, and categorized according to their localization on DNA (Table 6). Additionally, we correlated the VUS according to the transactivity reduction level [9] as well as the relative fitness score [10]. However it must be pointed out that Kotler et al. restricted their analysis to DBD variants [10] whereas only 9 out of 20 VUS detected in our cohort, were localized on the DNA-binding domain. Yet no difference could be identified concerning the distinct behavior of breast cancer manifestation.

**Table 6:** Functional data- variants of uncertain significance in TP53 gene

Genomic position	Protein change	DNA domain	Transcriptional activity in yeast (% of wild-type)	Relative fitness score (RFS)**
c.26G > A	p.Ser9Asn	Transactivation domain 1	(26.01%)	Unknown
c.29 T > G	p.Val10Gly	Transactivation domain 1	(98.91%)	Unknown
c.217G > A	p.Val73Met	Transactivation domain 2	(93.8%)	Unknown
c.255 T > C	p.Pro85Pro	Transactivation domain 2	Unknown	Unknown
c.266C > A	p.Pro89His	Transactivation domain 2	(5.96%)	Unknown
c.333G > T	p.Leu111Leu	DNA-binding domain	Unknown	RFS ≤ − 1
c.375 + 6 T > C		DNA-binding domain	Unknown	Unknown
c.457C > T	p.Pro153Ser	DNA-binding domain	(83.55%)	RFS ≤ − 1
c.470 T > G	p.Val157Gly	DNA-binding domain	(9.2%)	RFS > − 1
c.523C > T	p.Arg175 Cys	DNA-binding domain	(84.36%)	RFS ≤ − 1
c.529_546del	p.Pro177_Cys182del	DNA-binding domain	Unknown	Unknown
c.572-574del	p.Pro191del	DNA-binding domain	Unknown	Unknown
c.663G > A	p.Glu221Glu	DNA-binding domain	Unknown	RFS ≤ − 1
c.847C > T	p.Arg283Cys	DNA-binding domain	(85.49%)	RFS ≤ − 1
c.927C > T	p.Pro309 Pro	Nuclear localization domain	Unknown	Unknown
c.1014 C > T	p.Phe338Phe	Tetramerization domain	Unknown	Unknown
c.1014 C > T	p.Phe338Phe	Tetramerization domain	Unknown	Unknown
c.1079G > C	p.Gly360Ala		(78.04%)	Unknown
c.1163A > C	p.Gln388Ala	Regulatory domain	(112%)	Unknown
c.1171G > A	p.Asp391Asn	Regulatory domain	(94.24%)	Unknown

RFS ≤ − 1: retaining wtp53-like anti-proliferative functionality

RFS > − 1: disrupting functionality

Hotspot mutations: R175H, R273H, R248Q, R248W, R273C, R282W

**RTS (relative fitness score)

## Discussion

In this study we describe *TP53g* mutations in the context of families with hereditary breast and ovarian cancer. (Likely) pathogenic variants in *TP53* gene were present in 0.6% of the cohort. This is in line with previous studies, which describe a low *TP53g* mutation prevalence among women, who have had breast cancer panel testing [22–25].

For classic Li-Fraumeni families, a profound high-risk surveillance program has been implemented including a full-body magnetic resonance imaging (MRI) scan to be conducted annually beginning at early age (Table 7) [26, 27]. The examinations are associated with enormous psychological and physiological stress and discomfort, which raises the question if a more individualized surveillance may be more acceptable for families with hereditary breast and ovarian cancer without additional features indicative of LFS.

**Table 7:** Recommended LFS screening protocol (based on the Toronto protocol)

Children (birth to 18 years)	
General assessment	Complete physical examination and blood tests every 3–4 months (blood-test: * 17-OH-progesterone, total testosterone, dehydroepiandrosterone sulfate, Androstenedione, complete blood count, erythrocyte sedimentation rate, lactate dehydrogenase; 24h urine cortisol, if feasible)
Brain tumor	Annual brain MRI (first MRI with contrast; thereafter without contrast if previous MRI normal and no new abnormality)
Soft tissue and bone sarcoma	Annual whole body MRI
Adrenocortical carcinoma	US of abdomen and pelvis every 3–4 months
Adults	
General assessment	Complete physical examination and blood tests every 3–4 months (blood-test: * 17-OH-progesterone, total testosterone, dehydroepiandrosterone sulfate, Androstenedione, complete blood count, erythrocyte sedimentation rate, lactate dehydrogenase; 24h urine cortisol, if feasible)
Breast cancer (age 18 years onward)	Breast awareness (age 18 years onward) Clinical breast examination twice a year (age 20 years onward) Semi-annual breast sonography (age 20 years onward) Annual breast MRI screening (ages 20–75) Consider risk-reducing bilateral mastectomy
Soft tissue and bone sarcoma (age 18 years onward)	Annual whole body MRI US of abdomen and pelvis every 3–4 months
Brain tumor (age 18 years onward)	Annual brain MRI (first MRI with contrast; thereafter without contrast if previous MRI normal)
Gastrointestinal cancer (age 25 years onward)	Upper endoscopy and colonoscopy every 2–5 years
Melanoma (age 18 years onward)	Annual dermatologic examination

Adapted by Villani et al. [26, 27, 33]

Large scale surveillance protocol (based on the Toronto protocol) is recommended for individuals with a pathogenic *TP53* germline mutation, which is associated with enormously psychological discomfort. However the wide adoption of next-generation sequencing (NGS) panels has led to a considerably higher prevalence of *TP53* mutations in the context of hereditary breast and ovarian cancer, whereof many individuals with *TP53* mutations lack classic personal or family history of LFS-related cancers. The clinical challenge is to define a subgroup of *TP53-*mutation carriers for whom the screening recommendation should differentiate from the classic LFS-families

*MRI* magnetic resonance imaging, *US* ultrasound

Taking the GC-HBOC inclusion criteria into consideration, one of the highest mutation frequencies of (likely) deleterious *TP53g* mutations was seen in families with early onset of breast cancer (< 36 years. (1.1%)) (Table 3). However, the mutation prevalence in this subgroup was lower than most studies previously reported, which were largely limited by a smaller sample size [4–7, 28, 29] (Table 8). Yet, similar results were recently demonstrated in a nationwide cohort study from the Netherlands. Their analysis revealed a prevalence rate of 2.2% among early onset breast cancer *BRCA1/BRCA2* mutation negative patients, unselected for family history and 0.9% if there were no additional features indicative of LFS [30].

**Table 8:** *TP53* germline mutation prevalence among early onset breast cancer patients, tested negative for a *BRCA1/2* germline mutation

Study	Subject	N	Family history/personal history of multiple LFS-related tumors	*TP53* prevalance (%)
Lalloo et al. [4]	Breast cancer < 31 years*	82**	Unselected	4.9% (4/82)
Ginsburg et al. [29]	Breast cancer < 30 years	95	Unselected	0% (0/95)
Mouchawar et al. [5]	Breast cancer < 30 years*	43**	Unselected	4.7% (2/43)
Gonzalez et al. [6]	Breast cancer < 30 years	14	No cancer in first/second degree relatives	7.1% (1/14)
McCuaig et al. [7]	Breast cancer < 31 years	13	Did not meet classic LFS, LFL or Chompret 2009 criteria	7.7% (1/13)
Bougeard et al. [28]	Breast cancer < 30 years	Not reported	Did not meet Chompret 2009 criteria	6% (not reported)
Bakhuizen et al. [30]	Breast cancer < 30 years	233**	No sarcoma, brain tumor or ACC in family history; no second LFS-related tumor (other than breast cancer)	0.9% (2/233)

Adopted by Bakhuizen et al. [30]

*Population based cohort

**Subgroup of total study population

Even though our analysis additionally revealed a prevalence rate of 1.2% in families with breast and ovarian cancer, it should be noted that the cohort is underrepresented (n = 1) and therefore does not allow drawing conclusions about the impact of a (likely) deleterious *TP53g* mutation on ovarian cancer. According to literature, genetic predisposition for ovarian cancer is still highly controversial.

When considering families with the exclusive occurrence of female breast cancer, the analysis revealed that (likely) deleterious *TP53g* mutations as well as variants of uncertain significance were much more frequent in families with bilateral compared to families with unilateral breast cancer (*TP53mut*: 1.1% vs. 0.3%; *TP53-VUS*: 1.4% vs. 0.9%) (Table 4).

Aiming to advance our understanding regarding possible correlations between cancer risks and characteristics depending on the type of *TP53g* mutation, we assigned all *TP53*g variants (*n * = 31: (likely) pathogenic variants and VUS) accordingly to the DNA localization.

As presumed, variants in the DNA-binding domain tended to correlate with early onset of breast cancer as well as early development of contralateral breast cancer. In particular tumor development in childhood/early adulthood was exclusively associated with *TP53g* alterations located on the binding domain. To further categorize the impact of these *TP53g* variants, we enforced an alignment with high-resolution mutation analysis by Kato et al. [9] as well as relative fitness score by Kotler et al. [10]. *TP53g*-variants displaying strong and very strong transactivity reductions and RFS >  − 1 were predominately associated with early onset malignancies (except breast cancer). Whether personal or family history, malignancies in childhood/early adulthood solely presented if p53 functionality was very strongly reduced and a relative fitness score of > − 1 was given.

Interestingly, scoping the two pedigrees with a (likely) deleterious *TP53g* mutation that only fulfilled the HBOC criteria, the corresponding *TP53* mutations were located on the core DNA-binding domain. Yet, we gained decisive knowledge when considering information on functional analysis. First, we aimed to elucidate the transactivity function of p53 [9]. Even though transactivity data was missing for *pedigree 1* (c.375G > A (p.Thr125Th)) (Fig. 2, *pedigree #1*)), the likely deleterious variant c.542G > A (p.Arg181His) of *pedigree 2* was associated with a medium reduction in p53 activity (Fig. 2, *pedigree #2*)). Furthermore when comparing *pedigree 1* and *2* (Fig. 2, *pedigree #1,2)* to the functional impact analysis by Kotler et al. [10], we found a RFS ≤  − 1 for both pedigrees, which constitutes a retainment of wtp53-like-anti-proliferative functionality. We hypothesize that this reduced impact on *TP53* functionality may contribute to the attenuated phenotype.

Yet, as already mentioned, these mutations have also been reported in several individuals meeting either classic Li-Fraumeni or Chompret criteria. This disparate manifestation might additionally imply a co-occurrence of other genetic and epigenetic modifiers, which attenuates or enhances the effect of decreased p53 anti-proliferative functionality.

Within the current guidelines of the European Reference Network GENTURIS, Freybourg et al. argue for a phenotypic variability, which strongly endorses the existence of genetic and environmental modifying factors [31].

Our findings indicate it is crucial to define subgroups, such as breast cancer families, as shown in this work, in order to possibly individualize surveillance programs in the future.

In this study, we further intended to analyze differences between (likely) deleterious mutations and VUS in *TP53,* especially concerning the clinical manifestation.

There is a current lack of knowledge regarding VUS, especially when they are located outside of the core DNA-binding domain. In general, it remains to be seen to what extend variants of uncertain significance will be assigned to a similar functional restriction as it is already proven for known (likely) pathogenic *TP53g* gene mutations, especially those with a strong reduction in transactivity function or rather RFS >  − 1, e.g. the *TP53* variant c.470 T > G, p.Val157Gly found in our collective. Even if variants are classified as VUS at present, they are within an extensive process of dynamic change. To ensure a sustainable improvement in reclassification and focusing an enhancement of patient care, GC-HBOC has established a group of experts (VUS-task force) who reevaluate these variants of uncertain significance.

It is essential to collect data centrally and constantly reassess the existing evidence, in order to advance patient care in the long term.

However, our study needs to be interpreted in light of its limitations regarding a small number of *TP53g* mutation carriers. Therefore, our current analysis must be considered as a hypothesis-generating study. Further data are necessary to confirm these findings. Additionally, we need to gain more knowledge about genetic and non-genetic modifiers that influence *p53* function.

Within the GC-HBOC, we intend to examine *TP53g* mutations in a larger cohort of HBOC families nationwide. However due to the low incidence of *TP53*g mutations, a systematic international collaboration is becoming highly desirable to focus on this issue and define subgroups of *TP53g*-mutations in non-suggestive clinical situations.

Agreeing on existing evidence and current guidelines published by the European Reference Network GENTURIS [31] and based of a lack of consensus, the established surveillance program should currently be recommended to all carriers of a (likely) deleterious *TP53g* mutation irrespective of the pedigree.

## Conclusion

In this study we were able to define a clinical subgroup among our cohort of HBOC families, which phenotypically differentiates from the characteristic LFS-families. By applying a classification following functional data like transactivity reduction level [9] as well as relative fitness score [10], we determined discrepancies of *TP53* functionality that suited the attenuated phenotype. This is an approach that could be useful in developing individualized screening efforts for *TP53g* mutation carrier in HBOC families. Due to the low incidence of *TP53g* mutations, it is essential to heighten the perception on this topic and provide conditions for national/international collaborations. This might help providing directions for clinical recommendations in the future.

## Electronic supplementary material

Below is the link to the electronic supplementary material.

Electronic supplementary material 1 (PDF 369 kb)

## Data Availability

Individual participant data that underly the results reported in this article, will be available after deidentification (text, tables, figures). Data will be available beginning immediately following publication and ending 36 months following article publication. Data will be shared with researchers who provide a methodologically sound proposal to achieve aims in the approved proposal. There is no certain mechanism for data sharing applications. Proposals should be sent to the corresponding author by email.

## References

[1] Evans DG (2006). Malignant transformation and new primary tumours after therapeutic radiation for benign disease: substantial risks in certain tumour prone syndromes. J Med Genet.

[2] Schon K, Tischkowitz M (2018). Clinical implications of germline mutations in breast cancer: TP53. Breast Cancer Res Treat.

[3] Mai PL (2016). Risks of first and subsequent cancers among TP53 mutation carriers in the National Cancer Institute Li-Fraumeni syndrome cohort. Cancer.

[4] Lalloo F (2006). BRCA1, BRCA2 and TP53 mutations in very early-onset breast cancer with associated risks to relatives. Eur J Cancer.

[5] Mouchawar J (2010). Population-based estimate of the contribution of TP53 mutations to subgroups of early-onset breast cancer: Australian Breast Cancer Family Study. Cancer Res.

[6] Gonzalez KD (2009). Beyond Li Fraumeni Syndrome: clinical characteristics of families with p53 germline mutations. J Clin Oncol.

[7] McCuaig JM (2012). Routine TP53 testing for breast cancer under age 30: ready for prime time?. Fam Cancer.

[8] Wappenschmidt B (2020). Criteria of the German Consortium for Hereditary Breast and Ovarian Cancer for the Classification of Germline Sequence Variants in Risk Genes for Hereditary Breast and Ovarian Cancer. Geburtshilfe Frauenheilkd.

[9] Kato S (2003). Understanding the function-structure and function-mutation relationships of p53 tumor suppressor protein by high-resolution missense mutation analysis. Proc Natl Acad Sci U S A.

[10] Kotler E (2018). A Systematic p53 Mutation Library Links Differential Functional Impact to Cancer Mutation Pattern and Evolutionary Conservation. Mol Cell.

[11] Varley JM (1997). Germ-line mutations of TP53 in Li-Fraumeni families: an extended study of 39 families. Cancer Res.

[12] Varley JM (2001). Characterization of germline TP53 splicing mutations and their genetic and functional analysis. Oncogene.

[13] Warneford SG (1992). Germ-line splicing mutation of the p53 gene in a cancer-prone family. Cell Growth Differ.

[14] Bougeard G (2008). Molecular basis of the Li-Fraumeni syndrome: an update from the French LFS families. J Med Genet.

[15] Ruijs MW (2010). TP53 germline mutation testing in 180 families suspected of Li-Fraumeni syndrome: mutation detection rate and relative frequency of cancers in different familial phenotypes. J Med Genet.

[16] Silva AG (2012). Number of rare germline CNVs and TP53 mutation types. Orphanet J Rare Dis.

[17] Hettmer S (2014). Anaplastic rhabdomyosarcoma in TP53 germline mutation carriers. Cancer.

[18] Wasserman JD (2015). Prevalence and functional consequence of TP53 mutations in pediatric adrenocortical carcinoma: a children's oncology group study. J Clin Oncol.

[19] Malcikova J (2014). TP53 mutation analysis in clinical practice: lessons from chronic lymphocytic leukemia. Hum Mutat.

[20] Monti P (2011). Dominant-negative features of mutant TP53 in germline carriers have limited impact on cancer outcomes. Mol Cancer Res.

[21] Hekmat-Scafe DS (2017). Using yeast to determine the functional consequences of mutations in the human p53 tumor suppressor gene: An introductory course-based undergraduate research experience in molecular and cell biology. Biochem Mol Biol Educ.

[22] Buys SS (2017). A study of over 35,000 women with breast cancer tested with a 25-gene panel of hereditary cancer genes. Cancer.

[23] Moran O (2017). Revisiting breast cancer patients who previously tested negative for BRCA mutations using a 12-gene panel. Breast Cancer Res Treat.

[24] Susswein LR (2016). Pathogenic and likely pathogenic variant prevalence among the first 10,000 patients referred for next-generation cancer panel testing. Genet Med.

[25] Kapoor NS (2015). Multigene Panel Testing Detects Equal Rates of Pathogenic BRCA1/2 Mutations and has a Higher Diagnostic Yield Compared to Limited BRCA1/2 Analysis Alone in Patients at Risk for Hereditary Breast Cancer. Ann Surg Oncol.

[26] Kratz CP (2017). Cancer Screening Recommendations for Individuals with Li-Fraumeni Syndrome. Clin Cancer Res.

[27] Villani A (2011). Biochemical and imaging surveillance in germline TP53 mutation carriers with Li-Fraumeni syndrome: a prospective observational study. Lancet Oncol.

[28] Bougeard G (2015). Revisiting Li-Fraumeni Syndrome From TP53 Mutation Carriers. J Clin Oncol.

[29] Ginsburg OM (2009). The prevalence of germ-line TP53 mutations in women diagnosed with breast cancer before age 30. Fam Cancer.

[30] Bakhuizen JJ (2019). TP53 germline mutation testing in early-onset breast cancer: findings from a nationwide cohort. Fam Cancer.

[31] Frebourg T (2020). Guidelines for the Li-Fraumeni and heritable TP53-related cancer syndromes. Eur J Hum Genet.

[32] Mai PL (2012). Li-Fraumeni syndrome: report of a clinical research workshop and creation of a research consortium. Cancer Genet.

[33] Villani A (2016). Biochemical and imaging surveillance in germline TP53 mutation carriers with Li-Fraumeni syndrome: 11 year follow-up of a prospective observational study. Lancet Oncol.

