# Supplementary material for: TP53 germline mutations in the context of families with hereditary breast and ovarian cancer: a clinical challenge
Source: Arch Gynecol Obstet. 2020 Nov 27;303(6):1557–67. doi: 10.1007/s00404-020-05883-x (PMC8087555; doi:10.1007/s00404-020-05883-x)
Supplement: Supplementary file 1 — Electronic supplementary material 1 (PDF 369 kb) [file 404_2020_5883_MOESM1_ESM.pdf]

Supplement 1: Clinical characteristics- deleterious and likely deleterious mutations in *TP53* gene

Genomic position	Protein change	DNA domain	Transcriptional activity in yeast (% of wild-type)	Relative fitness score (RFS)**	LFS/Chompret criteria	Index	Tumor subtype	Family history
c.375G>A	p.Thr125Thr	DNA binding domain	Unknown	*RFS≤-1*	No	BC bilateral 39 yrs	HR pos, Her2 neg	none
c.542G>A	Arg181His	DNA binding domain	Medium reduction (50.3%)	*RFS≤-1*	No	BC 46yrs	HR pos, Her2 neg	mother: BC 41 yrs<br>aunt (m): unknown cancer 40yrs
c.700T>C	Tyr234His	DNA binding domain	Very strong reduction (0.89%)	*RFS> -1*	Yes (Chompret criteria (1))	BC 33yrs and CRC 21yrs	HR neg HER2 pos	brother: glioblastoma 32yrs
c.717C>G	Asn239Lys	DNA binding domain	Moderate strong reduction (26.4%)	*RFS< -1*	Yes (classic LFS)	BC bilateral 32&34 yrs	32yrs: HR pos HER2 pos; 34yrs: TNBC	brother: lung cancer 36yrs<br>sister: BC 26 yrs<br>sister: BC 39 yrs;<br>corresponding son: osteosarcoma 14 yrs and gastric cancer 35yrs<br>father: brain tumor <50yrs
c.733G>A	Gly245Ser	DNA binding domain	Strong reduction (4.2%)	*RFS> -1*	Yes (Chompret criteria (1))	BC 49yrs	HR pos, Her2 neg	sister: bilateral BC 30&45 yrs and CRC 55yrs<br>brother: ACC 56yrs, CRC 53yrs and prostate cancer 56 yrs;<br>father: testicular cancer 32 yrs, prostate cancer 68yrs and lung cancer 71 yrs<br>uncle (p): brain tumor 48yrs<br>aunt (p): pancreas cancer 59 yrs
c.742C>T	Arg248Trp	DNA binding domain	Very strong reduction (0.09%)	RFS>-1	Yes (Chompret criteria (4))	BC 26 yrs	HR pos, Her2 pos	none
c.743G>A	p.Arg248Gln	DNA binding domain	Very strong reduction (0.21%)	*RFS> -1*	Yes (classic LFS)	BC 34yrs and osteosarcoma 16yrs	TNBC	daughter: neuroblastoma 2yrs
c.783-2A>G splice acceptor		DNA binding domain	Unknown	Unknown	Yes (Chompret criteria (4))	BC 35yrs	HR pos, Her2 neg	sister: BC 35yrs<br>mother: BC: 28yrs<br>uncle (m): pharynx cancer 68yrs<br>grandmother (m): CRC 86 yrs
c.800G>C	p.Arg267 Pro	DNA binding domain	Very strong reduction (0.46%)	*RFS> -1*	Yes (Chompret criteria (4))	BC 22yrs	HR pos HER2 pos	none
c.817C>T	p.273Arg>Cys	DNA binding domain	Very strong reduction (0.76%)	*RFS> -1*	Yes (Chompret criteria (1))	BC 43 yrs	HR pos, Her2 pos	daughter: leukemia 4 yrs<br>brother: rectum cancer 17yrs<br>mother: BC 40 yrs and “abdominal cancer” 54 yrs<br>aunt (m): lung cancer 52 yrs

c.818G>A	Arg273His	DNA binding domain	Strong reduction (2.51%)	*RFS> -1*	Yes (Chompret criteria (4))	BC bilateral 28&32yrs	28yrs: TNBC; 32 yrs: HR pos, Her2 pos	mother: BC 28yrs

Supplement 2: Clinical characteristics- variants of uncertain significance in *TP53* gene

Genomic position	Protein change	DNA domain	Transcriptional activity in yeast (% of wild-type)	Relative fitness score (RFS)**	LFS/Chompret criteria	Index	Tumor subtype	Family history
c.26G>A	p.Ser9Asn	Transactivation domain 1	(26.01%)	Unknown	No	DCIS 49 yrs	-	father: melanoma 70 yrs
c.29T>G	p.Val10Gly	Transactivation domain 1	(98.91%)	Unknown	Yes (Chompret criteria (4))	BC 28 yrs	HR pos HER2 pos	None
c.217G>A	p.Val73Met	Transactivation domain 2	(93.8%)	Unknown	No	DCIS 46 yrs	-	mother: BC 43 yrs<br>cousin (m): DCIS 45 yrs
c.255T>C	p.Pro85Pro	Transactivation domain 2	Unknown	Unknown	Yes (Chompret criteria (4))	BC 30 yrs	N.A.	none
c.266C>A	p.Pro89His	Transactivation domain 2	(5.96%)	Unknown	No	BC 45 yrs	HR pos HER 2 neg	father: prostate cancer 62 yrs and pancreatic cancer 84 yrs
c.333G>T	p.Leu111Leu	DNA-binding domain	Unknown	*RFS≤-1*	No	BC 39 yrs	HR pos HER2 neg	mother: BC bilateral 48 & 68 yrs<br>father: CRC 51 yrs<br>grandmother (p): CRC 71 yrs
c.375+6T>C		DNA-binding domain	Unknown	Unknown	No	BC 45yrs	HR pos HER2 pos	sister: BC 41 yrs (negative for VUS TP53)<br>father: CRC 65 yrs and pancreatic cancer 77 yrs
c.457C>T	p.Pro153Ser	DNA-binding domain	(83.55%)	*RFS≤-1*	No	BC 39 yrs	HR pos HER2 neg	sister: BC 50yrs
c.470T>G	p.Val157Gly	DNA-binding domain	(9.2%)	*RFS>-1*	No	BC bilateral 33yrs	HR pos HER2 neg and HR neg HER2 pos	none
c.523C>T	p.Arg175 Cys	DNA binding domain	(84.36%)	*RFS≤-1*	No	BC 48 yrs	HR pos Her2 neg	mother: BC 48yrs
c.529_546del	p.Pro177_Cys182del	DNA binding domain	unknown	unknown	Yes (Chompret criteria (1))	BC bilateral 37&39yrs	37yrs: TNBC; 39yrs HR pos HER2 neg	sister: BC 32yrs<br>father: choroidal melanoma 72yrs
c.572-574del	p.Pro191del	DNA-binding domain	Unknown	Unknown	*Yes (Chompret criteria (1))*	DCIS 45 yrs	-	sister (twin): CRC 33 yrs<br>sister: brain tumor 32 yrs<br>mother: BC 53yrs and abdominal sarcoma 64yrs
c.663G>A	p.Glu221Glu	DNA-binding domain	Unknown	*RFS≤-1*	No	BC 64 yrs	TNBC	mother: BC 67 yrs
c.847C>T	p.Arg283Cys	DNA-binding domain	(85.49%)	*RFS≤-1*	No	BC 50 yrs	HR pos HER2 neg	father: plasmozytom 84yrs
c.927C>T	p.Pro309 Pro	Nuclear localization domain	Unknown	Unknown	No	BC bilateral 48&61yrs	BC 48yrs: HR pos HER2 neg; BC 61yrs: HR pos HER2 neg	father: gastric cancer 76 yrs<br>aunt (p): gastric cancer 75yrs
c.1014 C>T	p.Phe338Phe	tetramerization domain	Unknown	Unknown	No	DCIS 50 yrs	-	sister: BC bilateral 45 yrs<br>mother: BC bilateral 81 & 85 yrs
c.1014 C>T	p.Phe338Phe	tetramerizatio	Unknown	Unknown	No	BC 34 yrs	HR pos HER 2	none

		n domain					neg	
c.1079G>C	p.Gly360Ala		(78.04%)	Unknown	No	BC bilateral 51&63 yrs	BC 51yrs: HR pos Her2 neg; BC 63 yrs: HR pos HER2 pos	sister: melanoma 50 yrs brother: prostate cancer mother: pleural carcinosis (unknown primaries) 75 yrs
c.1163A>C	p.Gln388Ala	regulatory domain	(112%)	Unknown	Yes (Chompret criteria (4))	BC 27 yrs	HR pos HER2 pos	Confirmed maternal inheritance, broader cancer diseases are missing
c.1171G>A	p.Asp391Asn	regulatory domain	(94.24%)	Unknown	No	BC 37 yrs	HR pos HER2 neg	None, parents healthy (mother s.a. bilateral Adnexectomy at the age of 30yrs)
